# Effect of human papillomavirus (HPV) vaccination on HPV infection and recurrence of HPV related disease after local surgical treatment: A systematic review and meta-analysis

**DOI:** 10.1371/journal.pone.0312128

**Published:** 2024-12-31

**Authors:** Qinxue Cao, Yantao Hou, Chaoyang Wang, Juntao Yin

**Affiliations:** 1 Department of Obstetrics and Gynecology, Huaihe Hospital, Henan University, Henan, China; 2 Henan Technical Institute, School of Mechanical and Electrical Engineering, Zhengzhou, China; 3 Department of General Surgery, Huaihe Hospital, Henan University, Henan, China; 4 Department of Pharmacy, Huaihe Hospital, Henan University, Henan, China; Kirklareli Universitesi, TÜRKIYE

## Abstract

**Background:**

The prophylactic vaccines available to protect against infections by human papillomavirus (HPV) are well tolerated and highly immunogenic. This systematic review and meta-analysis aimed to explore the efficacy of HPV vaccination on the risk of HPV infection and recurrent diseases related to HPV infection in individuals undergoing local surgical treatment.

**Methods:**

A literature search was performed using PubMed/MEDLINE, Embase, the Cochrane Library, Scopus, Web of Science, and bioRxiv/medRxiv from inception to July 15, 2024. Randomized controlled trials (RCTs) reporting the effect of HPV vaccination on HPV infection and recurrence of HPV related disease after local surgical treatment vs no HPV vaccination were included. The primary outcome measure was risk of recurrence cervical high-grade squamous intraepithelial lesion (HSIL) after local surgical treatment, with follow-up as reported by individual studies. Included studies were assessed for risk of bias using the Revised Cochrane risk-of-bias (RoB 2.0 tool). Pooled risk ratios (RR) and 95% confidence intervals (CI) were calculated. No restrictions were applied on language, the date of publication, age, sex, and country. All analyses were carried out using the Review Manager 5 software (version 5.4).

**Results:**

Eight RCTs (n = 3068) met the inclusion criteria. The risk of cervical HSIL recurrence was not reduced in individuals who were vaccinated compared with those who were not vaccinated (RR 0.92, 95% CI: 0.66–1.27; *I*^*2*^ = 40%). However, HPV vaccination reduced the risk of recurrence of cervical HSIL related to the HPV types HPV16/18, but uncertainty was large (RR 0.57, 95% CI: 0.18–1.84; *I*^*2*^ = 29%).

**Conclusions:**

Adjuvant HPV vaccination after surgical excision is not associated with a reduced risk of recurrent HSIL overall or a reduced risk of recurrent lesions caused by the most oncogenic strains (HPV16/18). Therefore, HPV vaccination should not be considered for adjuvant treatment in patients undergoing surgical excision.

## Introduction

With the vaccination of human papillomavirus (HPV) vaccines, cervical cancer prophylaxis has been revolutionized [1]. Cervical cancer could be virtually eliminated by increasing HPV vaccines coverage and efforts to improve cervical cancer screening [2]. HPV vaccines can prevent HPV infection and related diseases in children before puberty, but cannot eliminate existing lesions and are ineffective against already existing lesions caused by HPV [3–7].

Women undergoing local surgery for cervical high-grade squamous intraepithelial lesion (HSIL) have been identified as a target high-risk population, and can benefit from vaccination with adjuvant vaccines to prevent cervical cancer. Cervical HSILs are the precursor condition to cervical cancer [8]. Patients with cervical HSIL on a cervical biopsy followed by a negative conization specimen are particularly susceptible to HPV infection and may be reinfected quickly [8,9]. These women are also at high risk of recurrent cervical HSIL and other malignancies associated with HPV infection [10–12], and repeated conisations may carry an increased risk of adverse reproductive outcomes [13–16].

The VENUS study demonstrates that HPV vaccination elicits a stronger immune response than natural infection [17]. Natural immunity from induced antibodies appears to weaken with the passage of time, while it was reported that vaccines can provide protection against reinfection or reactivation in seropositive individuals with prior clearance of the infection [18–20]. This vaccination may confer beneficial effects against new infections and reinfections from the same HPV type shortly after treatment, although this effect is unlikely to promote the clearance of an established infection in isolation. The potential of the vaccine to enhance the efficacy of local surgical treatment and promote viral clearance remains unknown.

Evidence of the efficacy of vaccination against human papillomavirus after conization is inconsistent. Two randomized controlled trials (RCTs) showed a reduced recurrence rate of cervical HSIL [21,22]. However, other studies reached contrary conclusions and suggested no benefit [23–25].

This meta-analysis aimed to assess the effect of HPV vaccines on the risk of HPV infection and recurrence of preinvasive disease related to HPV infection after local surgical treatment for cervical diseases.

## Materials and methods

### Protocol and registration

This systematic review was registered with the International prospective register of systematic reviews (PROSPERO, CRD42024512507), and was conducted in strict accordance with the recommendations of the Cochrane Handbook [26] and the updated Preferred Reporting Items for Systematic Reviews of Interventions (PRISMA) guidelines [27].

### Eligibility criteria

When the outcomes of the same studies were reported in different publications, the study with the largest sample size was enrolled in this meta-analysis. Inclusion criteria were predetermined and organized in accordance with the PICOS acronym: Participants (P): patients with cervical HSIL who have undergone local surgical treatment; Intervention (I): HPV vaccine; Control (C): no HPV vaccine; Outcomes (O): recurrence of cervical HSIL after local surgery related to HPV; recurrence of cervical LSIL, and cervical HSIL related to HPV16/18 types; and HPV infections after treatment; and Study design (S): included only RCTs. Cohort studies, matched case-control series, reviews, and studies lacking of information on outcomes, were excluded. No restrictions were imposed on language, the date of publication, age, sex, and country.

The study included all RCTs that reported HPV infection rates or recurrence of diseases related to HPV infection after local surgery for genital diseases related to HPV in vaccinated individuals. And only those studies that also reported the results of an unvaccinated cohort as control group were enrolled in this review. No restrictions were imposed on language, region, publication time, participant demographics, or publication status. Studies were enrolled independent of surgical technique, type of vaccine, and timing of vaccination.

### Search strategy

A comprehensive literature search was conducted in PubMed/Medline, EMBASE, the Cochrane Library databases, Scopus, Web of Science, and bioRxiv/medRxiv (from inception to July 15, 2024). Two independent reviewers (QXC and JTY) formulated the search strategy and screened the studies. The Cochrane-validated filter for randomized controlled trials was applied [28]. The search strategy was adjusted based on descriptors in each database. In addition, references of all included studies were also manually searched to determine any potentially qualified studies.

### Study screening and selection

Two authors (Q.X.C. and C.Y.W.) performed independently the screening and selection of the studies. The inconsistencies were resolved by a third author (J.T.Y.). The first step was to import the research papers from the designated database into EndNote X20 and deleted the duplicate items. After excluding irrelevant studies by evaluating their titles and abstracts, the full texts of the studies were read. If the information of studies located in databases was incomplete, the corresponding author was contacted by data extractors (J.T.Y. and C.Y.W.) via email for relevant information.

### Data extraction

If the studies met the eligibility criteria, data were extracted by two independent investigators (Q.X.C. and C.Y.W.) in a prespecified data extraction form. The data extraction form included the following items: the first author’s name, the publication year, the region in which the study was conducted, the mean age of the participants, type of vaccine, Vaccination timing, Follow-up duration, outcomes, recurrence, and associated factors were all included in the data extraction form.

### Quality assessment

Risk of bias was evaluated by two investigators independently (QXC and CYW) according to the updated Cochrane risk-of-bias tool (RoB-2.0 tool) for randomized controlled trials [29,30]. Disagreements between investigators were discussed or solved by a third investigator (JTY).

### Definitions of outcomes

The primary outcome was recurrence of cervical HSIL after local surgery related to HPV. Secondary outcomes included cervical HSIL related to HPV16/18 types, recurrence of cervical LSIL, and HPV infections after treatment.

### Statistical analysis

The data from randomized controlled trials were pooled, which reported the effect of vaccination shortly before, at the time of, or within 12 months after the local surgery. We used crude data for the primary outcome, and sensitivity and subgroup analyses.

We estimated each pooled effect size by using risk ratio (RR) and corresponding 95% confidence interval (CI) with a fixed- or random- effect model based on the Mantel-Haenszel method [31]. Statistical heterogeneity was evaluated using the χ2 test (*P*<0.10) and was quantified using *I*^*2*^ [32,33].

When more than 10 studies reported a certain outcome, visual inspection of the funnel plot and Egger’s test were used to evaluate the effects of publication bias [34]. The method of “trim and fill” was adopted to assess the effect of publication bias if publication bias was detected.

To identify possible sources of heterogeneity, a leave-one-out sensitivity analysis [31] was conducted for the primary outcome (risk of recurrence of cervical HSIL after local surgery). A series of subgroup analyses were also carried out to identify sources of heterogeneity and differences in pooled estimates for the primary outcome (risk of recurrence of cervical HSIL after local surgery) based on timing of vaccination (up to three months before *vs* at the time of or up to 12 months after treatment) and vaccine type (Cervarix *vs* Gardasil *vs* Gardasil-9). All statistical analyses were carried out with the Review Manager 5 software (version 5.4).

## Results

### Characteristics of the included studies

**Fig 1:**
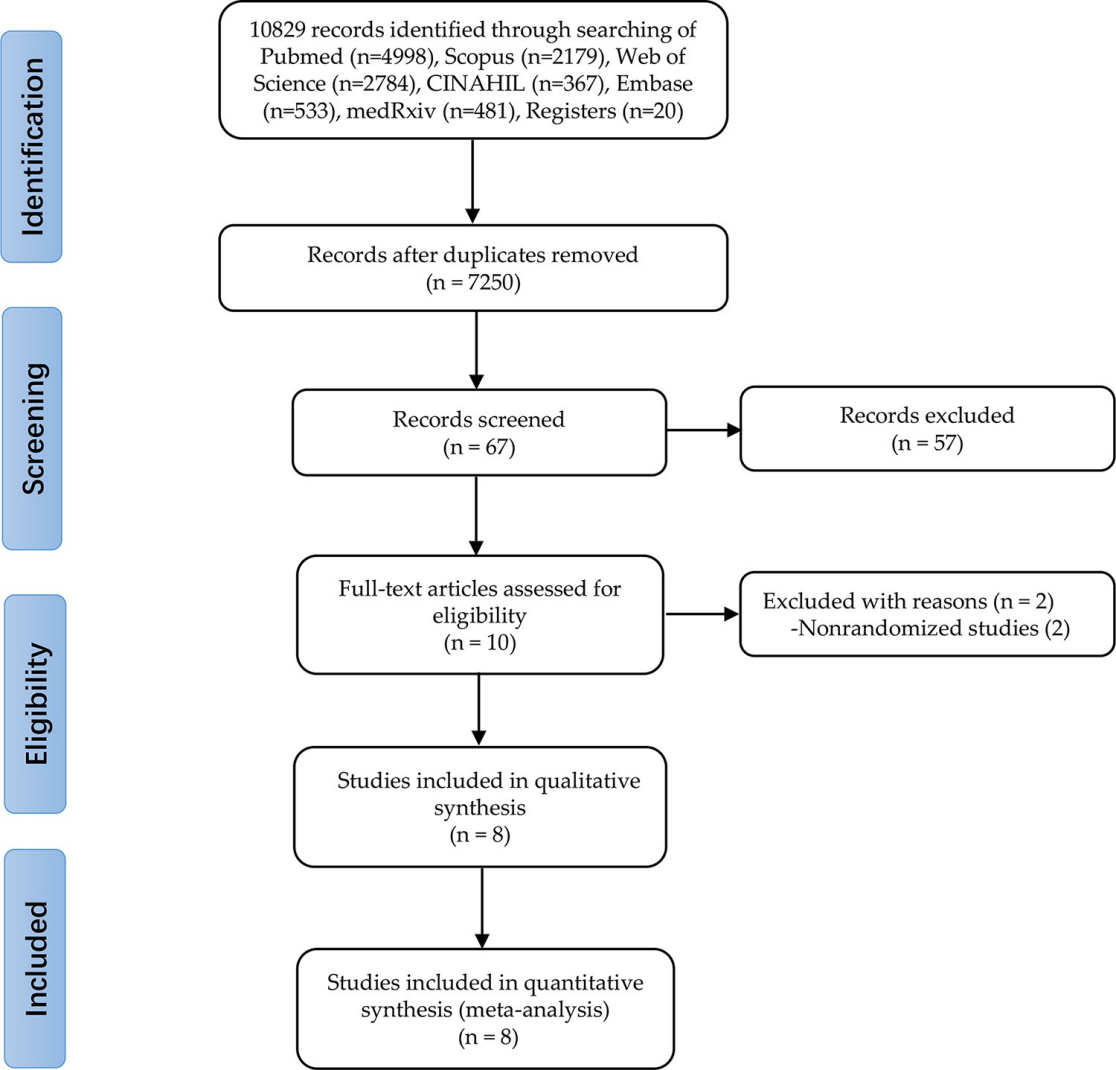
Research selection procedure based on the PRISMA flowchart.

**Fig 2:**
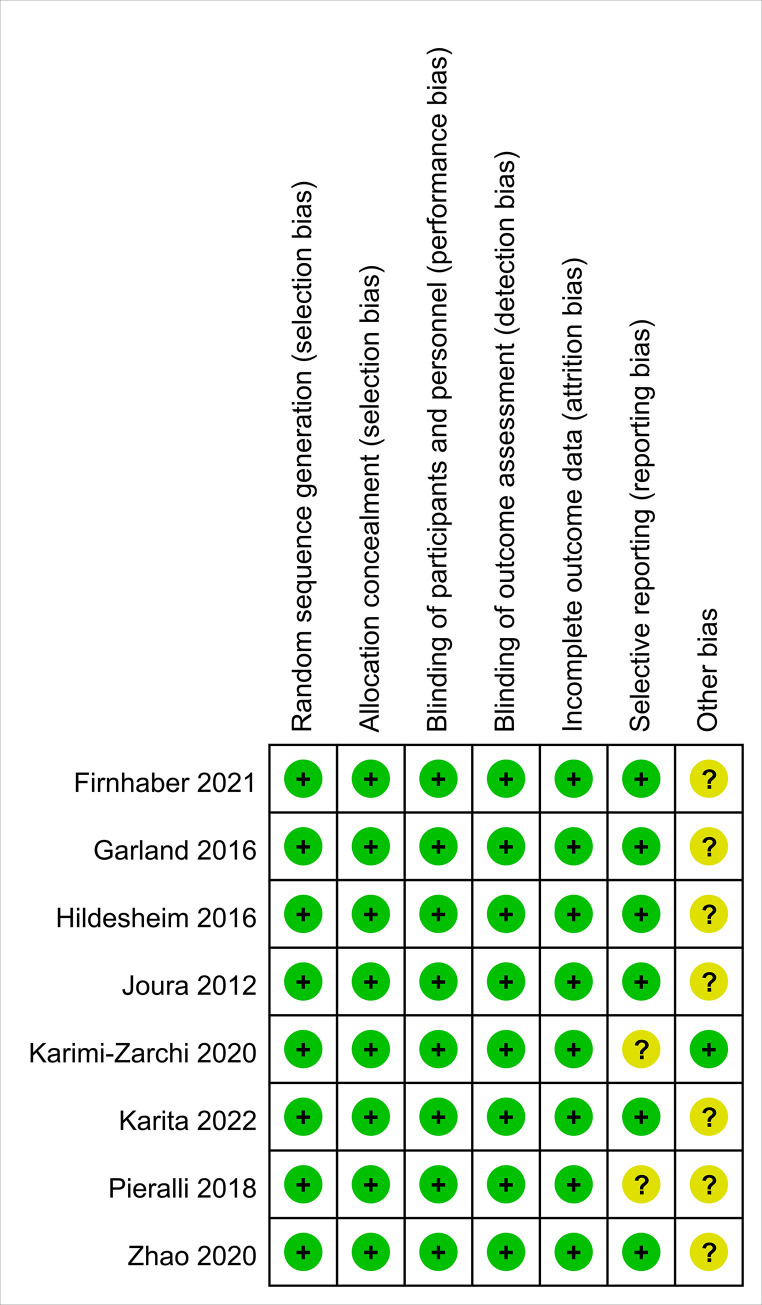
Risk of bias assessment was performed using the Cochrane Risk of Bias RoB2.0 tool.

There were 11362 studies were identified. All of them were screened for inclusion by title and abstract assessment, and 10 full-text articles were screened for eligibility. No other studies were retrieved from the reference lists of enrolled studies. Of these, 2 were ruled out because of the study design (not RCT) [35,36]. Thus, 8 studies with 3068 participants, all peer-reviewed studies, met the criteria for inclusion and were included into the review (Fig 1; Table 1) [21,22,24,25,37–40]. All of the 8 studies were assessed as low risk of bias according to the revised Cochrane RoB-2.0 tool (Fig 2).

**Table 1:** Characteristics and outcomes of studies included in this study.

Study ID	Country	Population	Intervention	Comparison	Vaccination timing	Follow-up duration	Outcomes†	Recurrence		
								HSIL	HSIL related to HPV16/18	LSIL
Firnhaber et al, [37] 2021	South Africa	180 women living with HIV with 1 year of follow-up [Vaccine group 90 –Non-vaccine Group 90] Age: 39.2 (mean) [Vaccine Group 40.1 –Non-Vaccine Group 39.1] CIN grade: 86 CIN2, 93 CIN3, 1 Inadequate [Vaccine group: 40 CIN2, 46 CIN3, 1 Inadequate–Non-Vaccine Group 49 CIN2, 44 CIN3]	LEEP	Quadrivalent vaccine [3 doses] *vs* No HPV vaccination	1 months Before treatment	26 months (minimum)	CIN2 CIN3 HSIL	28/87:27/87	-	-
Garland et al, [38] 2016	Multi-national (14 countries)	454 women treated for cervical lesions [Vaccine group 190 –Non-vaccine Group 264] Age: N/A	LLETZ, Conization	Bivalent vaccine [doses N/A] *vs* Hepatitis A vaccine	19.1 (1.5–46.5) and 26.5 (0.8–48.3) months before the treatment	47.3 (median)	CIN1+ CIN2+ HSIL LSIL CIN1+ HPV 16–18 CIN2+ HPV 16–18 CIN1 CIN2 Abnormal cytology VIN/VaIN 1+ VIN/VaIN 2+	1/159:4/215	0/174:1/234	27/101:21/110
Hildesheim et al, [39] 2016	Costa Rica	311 women treated for high grade cervical disease [Vaccine group 142 –Non-vaccine Group 169] Age: N/A CIN Grade: 154 normal, 67 LSIL, 87 HSIL [Vaccine group 1 Inadequate, 57 Normal, 36 LSIL, 47 HSIL–Non-vaccine group 1 Inadequate, 97 Normal, 31 LSIL, 40 HSIL]	LLETZ	Bivalent vaccine [3 doses 80%, 2 doses 12.4%, 1 dose 7.4%] *vs* Hepatitis A vaccine	28.2 months (median) Before treatment	27.3 months (median)	CIN2+ * CIN2+ HPV 16–18 * HSIL HSIL+ HPV 16–18 * LSIL Abnormal cytology Persistent HPV Infection Incident HPV infection	10/142: 4/169	3/142:2/169	33/142: 31/169
Joura et al, [40] 2012	Multi-national (13 countries)	1350 women treated for cervical disease [Vaccine group 587 –Non-vaccine Group 763] Age: 19.8 (mean) [Vaccine group 19.9 –Non-vaccine Group 19.8] CIN grade: 113 ASCUS, 23 ASCH, 232 LSIL, 65 HSIL [Vaccine group 47 ASCUS, ASCH 13, 112 LSIL, 36 HSIL Non vaccine group 65 ASCUS, 10 ASCH, 120 LSIL, 29 HSIL]	LLETZ (84.7%), Conization (13%), Cryotherapy (0.7%), Other (2.1%)	Quadrivalent vaccine [585/587 3 doses, 2/587 2 doses] *vs* 225 g aluminum Hydroxyphosphate sulfate	Before the treatment	44 months (maximum)	CIN1+ CIN2+ CIN1+ HPV 16–18 CIN2+ HPV 16–18 CIN1 CIN2 CIN3 VIN/VaIN 1+ VIN/VaIN 2+ Genital warts	-	-	-
Karimi-Zarchi et al, [20] 2020	Iran	242 women treated for CIN1 or high grade CIN (CIN2-3) [Vaccine group 138 –Non-vaccine Group 104] Age: 32.59 (mean) [Vaccine Group 31.7 –Non-Vaccine Group 33.04] CIN grade: 80 CIN1, 85 CIN2, 77 CIN3 [Vaccine group: 45 CIN1, 50 CIN2, 43 CIN3 –Non-Vaccine Group 35 CIN1, 35 CIN2, 34 CIN3]	LLETZ, CKC, Ablation	Quadrivalent vaccine [35/138 2 doses, 103/138 3 doses] *vs* No HPV vaccination	At the time of treatment	24 months (minimum)	CIN1+ CIN2+ CIN1 ICC	-	-	-
Karita et al, [23] 2022	United States	187 women who were previously treated for anal or vulvar HSIL and HSIL-free [Vaccine group 104 –Non-vaccine Group 83] Age: 55 (mean) [Vaccine Group 30.1 –Non-Vaccine Group 29.8] HSIL grade: 187 [Vaccine group: 104 Normal Non-Vaccine Group 83 Normal]	NI	9vHPV [3 doses] *vs* No HPV vaccination	Before the treatment	48 months (maximum)	HSIL	15/104: 18/83	-	-
Pieralli et al, [19] 2018	Italy	178 women treated for CIN with negative HPV test, cytology, and colposcopy 3 months after treatment [Vaccine Group 89 –Non-vaccine Group 89] Age: 32 (mean) [Vaccine Group 32.1—Non-vaccine Group 31.8] CIN grade: 30 LSIL, 148 HSIL	N/A	Quadrivalent vaccine [3 doses] *vs* No HPV vaccination	3 months after treatment	36 months (minimum)	CIN1+ CIN2+ HSIL HSIL+ HPV 16–18 LSIL CIN1+ HPV 16–18 CIN2+ HPV 16–18 CIN1 Abnormal cytology VIN/VaIN 1+ VIN/VaIN 2+	0/89:4/89	0/89:4/89	3/89:8/89
Zhao et al, [22] 2020	China	166 women treated for cervical lesions [Vaccine group 86 –Non-vaccine Group 80] Age: 18–25	LLETZ, Conization	Bivalent vaccine *vs* Aluminum hydroxide	17 months (median) before the treatment	50 months (median)	CIN1+ CIN2+ HSIL HSIL+ HPV 16–18 LSIL CIN2+ HPV 16–18 CIN1 CIN2 CIN3 VIN/VaIN 1+ VIN/VaIN 2+	1/80: 1/73	0/80: 0/73	2/80: 1/73

*median (range), #mean (SD)

† The outcome was confirmed by histology in most of the studies, * Describes studies were outcome was determined by either histology or cytology.

*AIN* anal intraepithelial neoplasia, *CIN* cervical intraepithelial neoplasia, *CKC* cold knife conisation, *HPV* human papilloma virus, *HSIL* high-grade squamous intraepithelial neoplasia, *ICC* invasive cervical cancer, *LLETZ* large loop excision of the transformation zone, *LSIL* low-grade squamous intraepithelial neoplasia, *MSM* men who have sex with men, *N/A* not applicable, *RCT* randomised controlled trial, *VIN/VaIN* vulvar intraepithelial neoplasia/vaginal intraepithelial neoplasia.

### Recurrence of cervical HSIL

**Fig 3:**
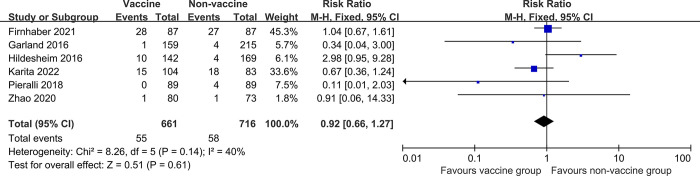
Forest plots assessing risk of recurrence of cervical HSIL between human papillomavirus (HPV) vaccinated and non-vaccinated groups after local conservative treatment for cervical intraepithelial neoplasia, irrespective of HPV type.

The analysis for risk of recurrence of cervical HSIL after local surgery showed that no significant difference was observed in RR of cervical HSIL recurrence between vaccinated participants at the time of treatment and unvaccinated participants (6 studies, 1376 participants; RR 0.92, 95% CI: 0.66–1.27, *I*^*2*^ = 40%) (Fig 3). Sensitivity analysis (one-by-one elimination method) showed that all of the six results remained statistically no significant (Table 2).

**Table 2:** Sensitivity analysis of Crude Risk Ratio (RR) Associated for cervical HSIL recurrence between participants who were vaccinated at the time of treatment and those who were unvaccinated.

Leave-one-out sensitivity analysis for cervical HSIL recurrence				
Estimates for Crude RR				
Removed study	RR	95% Lower CI	95% Upper CI	*p*
Firnhaber et al. 2021 [37]	0.98	0.76	1.27	= 0.10
Garland et al. 2016 [38]	1.02	0.82	1.28	> 0.10
Hildesheim et al. 2016 [39]	0.92	0.73	1.16	> 0.10
Karita et al. 2022 [23]	1.09	0.86	1.38	> 0.10
Pieralli et al. 2018 [19]	1.04	0.83	1.30	> 0.10
Zhao et al. 2020 [22]	1.00	0.80	1.25	= 0.10
**Overall**	**1.00**	**0.80**	**1.25**	**> 0.10**

**Fig 4:**
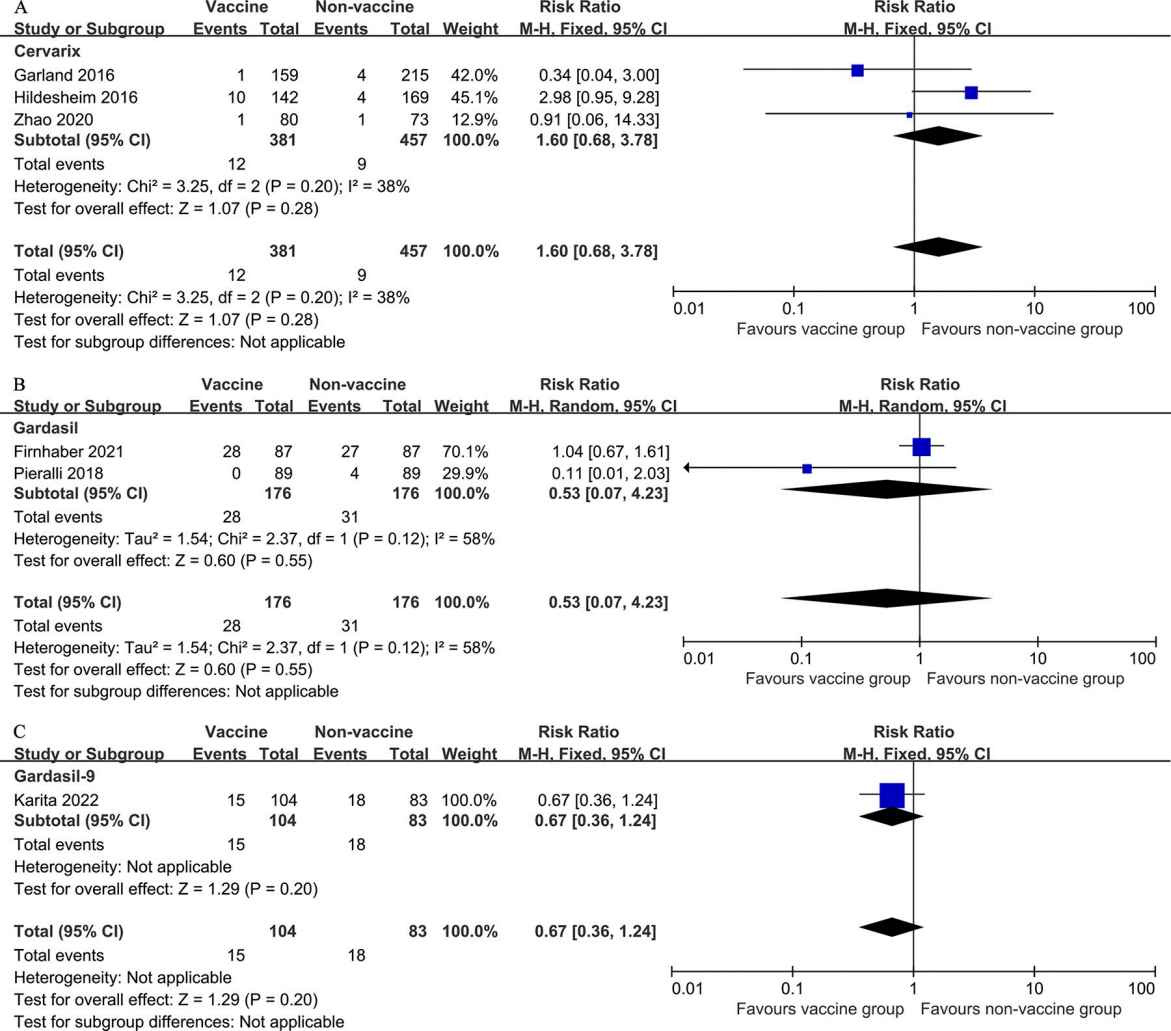
Forest plots demonstrating the subgroup analyses for the primary outcome (HSIL recurrence after local surgical treatment for cervical intraepithelial neoplasia) based on vaccine type.

Subgroup analysis was conducted based on type of vaccine. No significant difference in RR of recurrence of cervical HSIL between the two groups in studies that used the Cervarix vaccine (three studies, 838 participants; RR 1.60, 95% CI: 0.68–3.78, *I*^*2*^ = 38%), the Gardasil vaccine (two studies, 352 participants; RR 0.53, 95% CI: 0.07–4.23, *I*^*2*^ = 58%), or the Gardasil-9 vaccine (one studies, 187 participants; RR 0.67, 95% CI: 0.36–1.24; Fig 4).

**Fig 5:**
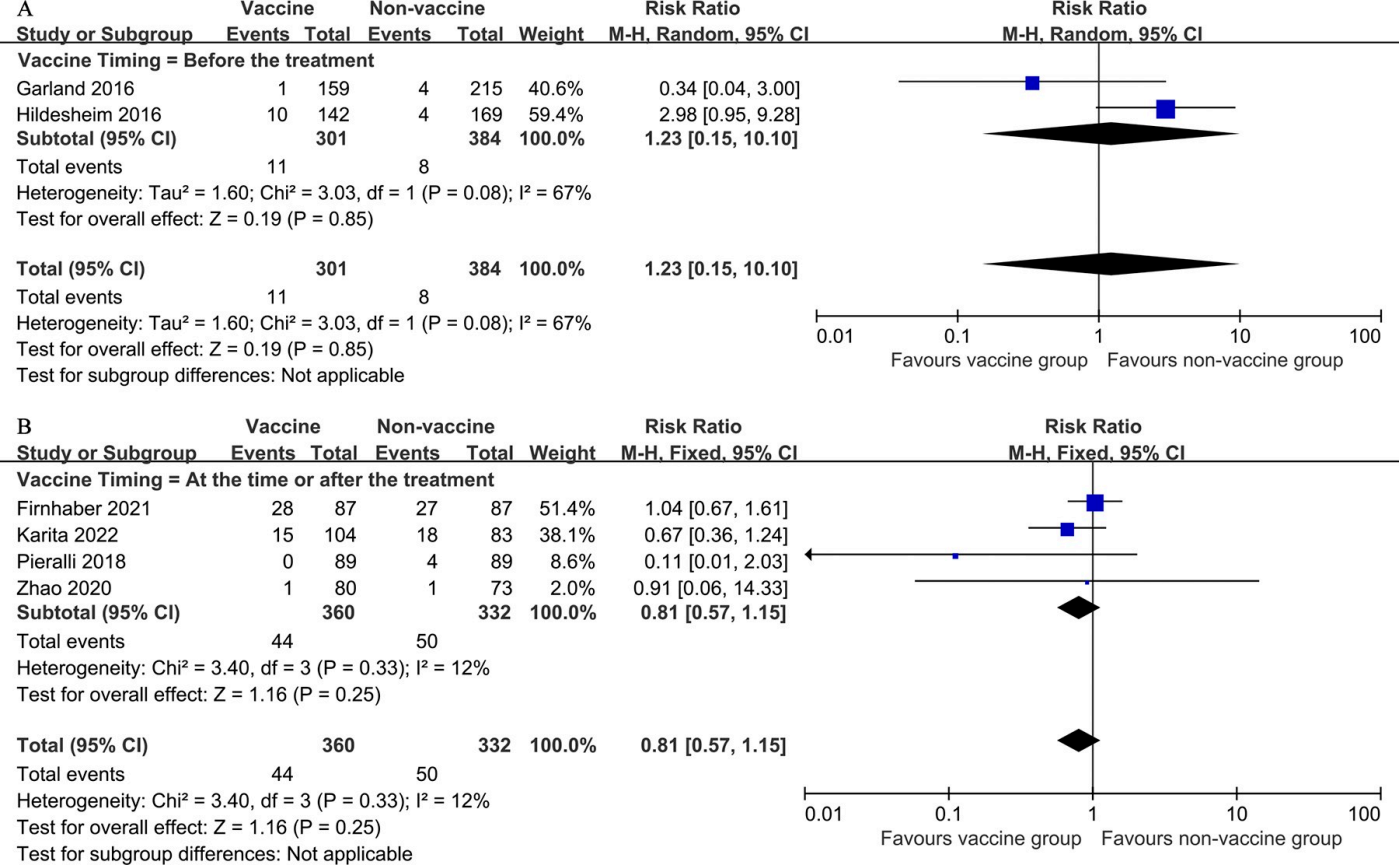
Forest plots demonstrating the subgroup analyses for the primary outcome (HSIL recurrence after local surgical treatment for cervical intraepithelial neoplasia) based on vaccination timing. A, Before the treatment. B, At the time or after the treatment.

Another subgroup analysis was carried out by timing of vaccination. Vaccination before treatment did not reduce the risk of recurrence of cervical HSIL, (2 studies, 685 participants; RR 1.23, 95% CI: 0.15–10.10, *I*^*2*^ = 67%; Fig 5A), nor did vaccination at the time of or after treatment reduce the risk of cervical HSIL recurrence despite the high degree of uncertainty (4 studies, 692 participants; RR 0.81, 95% CI: 0.57–1.15; *I*^*2*^ = 12%; Fig 5B).

### Recurrence of cervical HSIL related to HPV16/18

**Fig 6:**
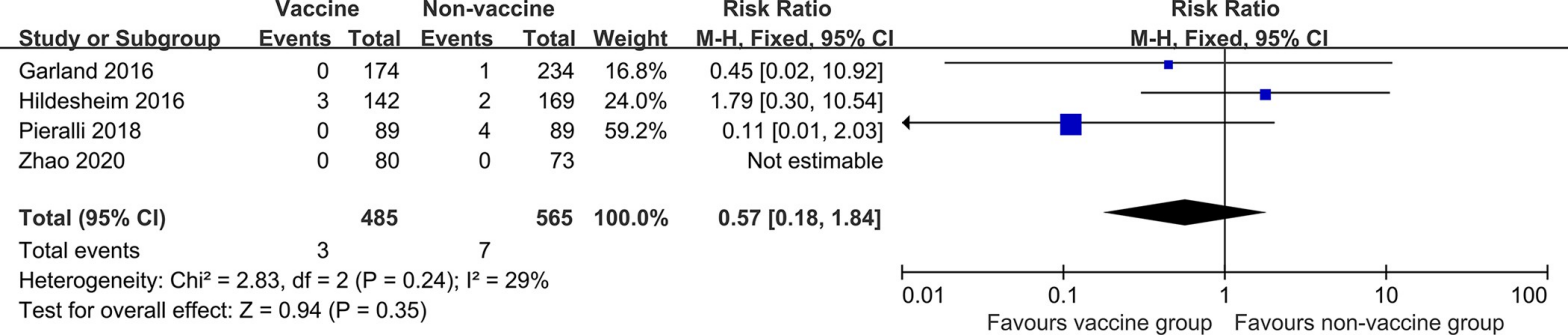
Forest plots assessing risk of recurrence of cervical HSIL between human papillomavirus (HPV) vaccinated and non-vaccinated groups after local treatment for cervical intraepithelial neoplasia, related to HPV types HPV16/18.

HPV vaccination did not reduce the risk of cervical HSIL recurrence related to HPV16/18 (4 studies, 1050 participants; RR 0.57, 95% CI: 0.18–1.84, *I*^*2*^ = 29%; Fig 6).

### Recurrence of cervical LSIL

**Fig 7:**
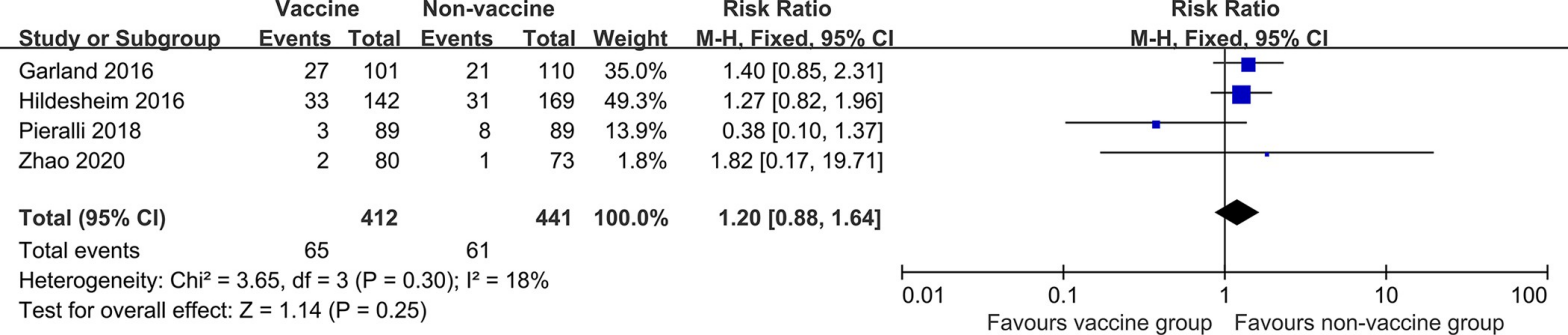
Forest plots assessing risk of recurrence of cervical LSIL between human papillomavirus (HPV) vaccinated and non-vaccinated groups after local treatment for cervical intraepithelial neoplasia, irrespective of HPV type.

No significant difference in RR of cervical LSIL recurrence between participants who were vaccinated at the time of treatment and unvaccinated participants (four studies, 853 participants; RR 1.20, 95% CI: 0.88–1.64, *I*^*2*^ = 18%; Fig 7).

### Publication bias

**Fig 8:**
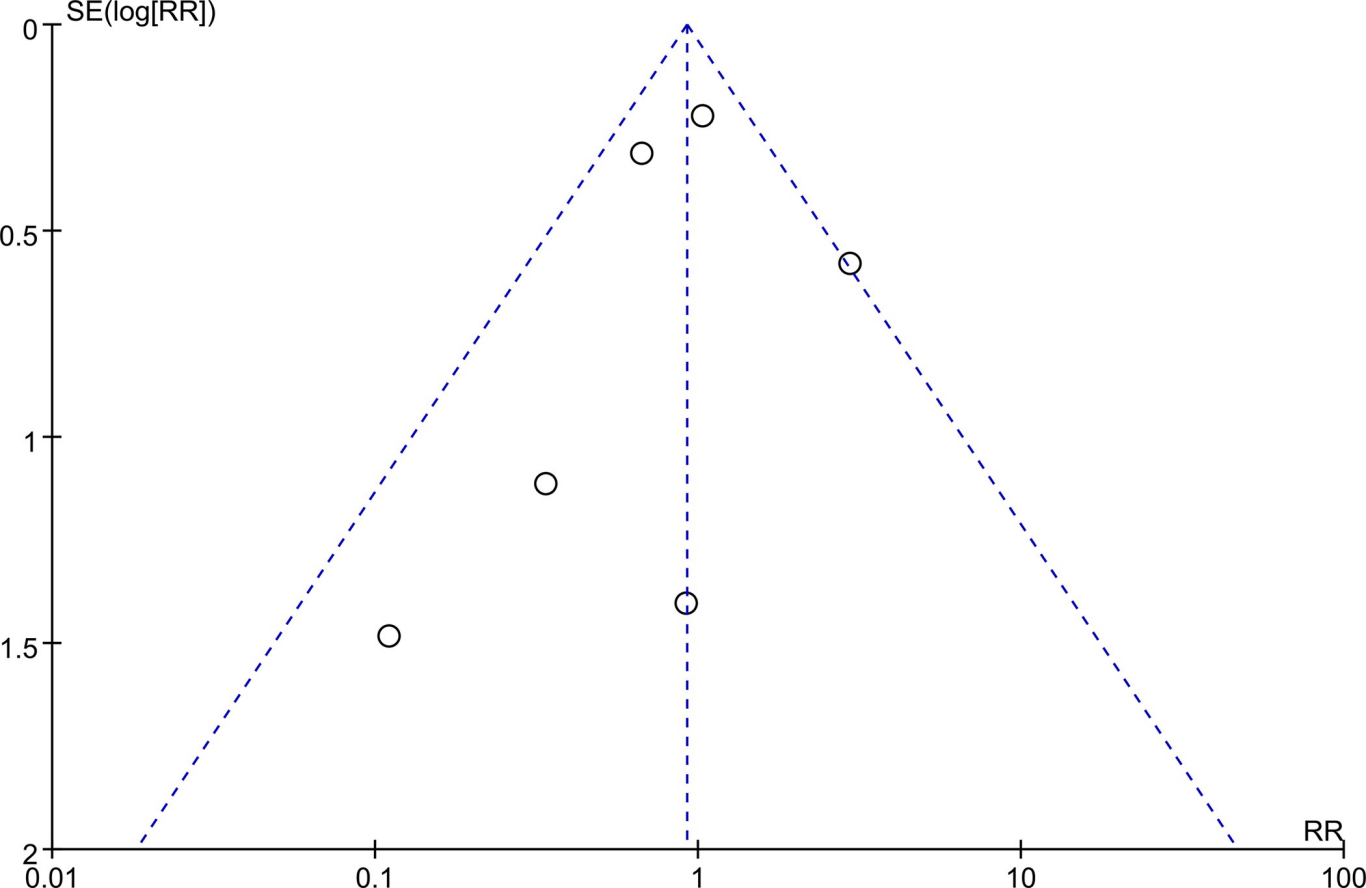
Funnel plot of studies examining cervical HSIL recurrence for the assessment of publication bias.

The funnel plot exhibited no asymmetry of studies examining HSIL recurrence for the assessment of publication bias, which indicates that there was no publication bias detected in our study (Fig 8).

## Discussion

The findings of this review indicate that adjuvant HPV vaccination at the time of local excision for cervical intraepithelial neoplasia (CIN) cannot reduce the risk of recurrence of cervical HSIL or LSIL. Moreover, HPV vaccination cannot reduce the risk of recurrence of cervical HSIL related to the HPV16/18. In studies that used the vaccine Cervarix, Gardasil, or Gardasil-9 respectively, no significant difference was shown in RR of recurrence of cervical HSIL between the two groups. Vaccination before treatment did not reduce the risk of recurrence of cervical HSIL, nor did vaccination at the time of or after treatment reduce the risk of cervical HSIL recurrence. Therefore, HPV vaccination should not be considered for adjuvant treatment in patients receiving local surgical treatment. The potential reasons for the observed lack of efficacy include differences in the timing of vaccination, the participants studied, or the nature of surgical treatment, which may also be the reason for heterogeneity.

To the best of our knowledge, our study is the first meta-analysis of RCTs to evaluate the effect of adjuvant HPV vaccination on the risk of cervical HSIL recurrence in individuals treated with local surgery. Whilst CIN2/3 is widely accepted as a surrogate marker for cervical cancer vaccine efficacy in studies of prophylactic or therapeutic HPV vaccines [41,42], and is generally be incorporated in HSIL according to the Bethesda System for Reporting Cervical Cytology (TBS), it is a poor predictor of progression [43,44]. After implementation of the new guidelines, conservative management became more frequent, and is now used for more than half of women with CIN2 [44]. Therefore, in this review, we chose recurrence of cervical HSIL rather than CIN2/3 after local surgical treatment as the primary outcome.

Up to now, there have been eight meta-analyses attempting to pool the evidence [45–52]. However, our findings on pooled estimates were in inconsistent with the outcomes of these meta-analyses, and our analysis sparked worries about data quality. All of the eight studies focused primarily on the CIN2/3 recurrence after local surgery, rather than cervical HSIL. Additionally, these studies included case control and cohort studies in addition to randomized controlled trials.

Our meta-analysis comprehensively assessed the effect of adjuvant HPV vaccination after surgical excision for cervical and other non-cervical diseases related to HPV infection, and rigorously assessed the risk of bias and heterogeneity. This analysis only enrolled RCTs in which vaccines were administered at the time of or after local surgery, which made potential inaccurate assessment of effect estimates eliminated and lowered heterogeneity. We examined the quality of the studies enrolled in our meta-analysis using the updated Cochrane RoB 2.0 tool. We also assessed the publication bias in this meta-analysis, and conducted analyses irrespective of HPV type and analyses for HPV16/18. We also performed two subgroup analyses based on timing of vaccination and type of vaccine. The results of which were basically consistent with the overall effect, proving the robustness of the conclusions.

However, the findings of our study should be interpreted with caution, as this study also has several limitations. Firstly, vaccinated participants might be younger than unvaccinated individuals, and the increased risk of disease recurrence may be partly due to older age. Secondly, confounding factors (such as smoking and drinking) that are associated with a higher risk of recurrence were not controlled in some studies. Thirdly, in each study, participants were vaccinated at different time points before or after local treatment for cervical intraepithelial neoplasia. Fourthly, the time period during which the enrolled studies were conducted might lead to bias because the staging system, terms and definitions may change with the passage of time. The enrolled studies were carried out over a long period, and populations can be recruited using the Bethesda or recently evolving ASCCP terminology. Finally, variability in length of follow-up (follow-ups of all the 8 enrolled studies were 24–64 months), diagnostic methods, timing of HPV vaccination, and type of HPV vaccine across studies might affect the accuracy of the effect estimates. The risk of cervical HSIL recurrence was lower with a median follow-up of >24 months, despite large uncertainty. Although the most frequent recurrence occurred within the first 24 months [53], the uneven length of follow-up is inherent defect of this study and similar studies; moreover, future disease episodes may not be associated with the originally, treated disease and confound estimates of (potential) vaccine impact. Therefore, large scale, high quality randomized controlled trials with follow-up ≥24 months are needed to provide more definitive evidence for the effect of HPV vaccination on the risk of HPV infection and recurrent diseases related to HPV infection in individuals undergoing local surgical treatment.

## Conclusions

In conclusion, adjuvant HPV vaccination after local surgical treatment for CIN is not associated with a reduced risk of HSIL recurrence overall or a reduced risk of recurrent lesions due to the most carcinogenic strains (HPV16/18). Therefore, HPV vaccination should not be considered for adjuvant treatment in patients receiving local surgical treatment.

## Supporting information

S1 Checklist PRISMA 2020 checklist. (DOCX)

S1 File (XLSX)

S1 Graphical abstract (TIF)
