# Supplementary figures and images for: Effect of human papillomavirus (HPV) vaccination on HPV infection and recurrence of HPV related disease after local surgical treatment: A systematic review and meta-analysis
Source: PLoS One. 2024 Dec 31;19(12):e0312128. doi: 10.1371/journal.pone.0312128 (PMC11687797; doi:10.1371/journal.pone.0312128)

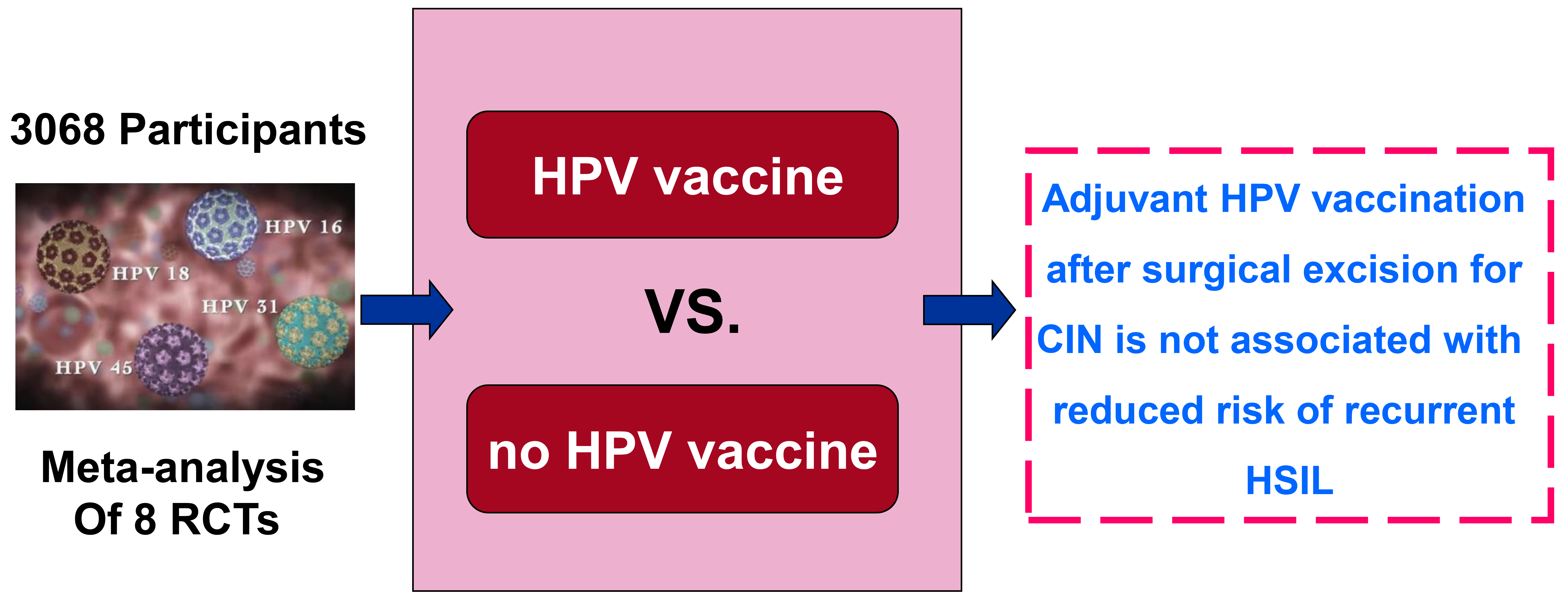

Supplement: S1 Graphical abstract — (TIF) [file pone.0312128.s003.tif]
